# Smartphone-based secondary prevention intervention for university students with unhealthy alcohol use identified by screening: study protocol of a parallel group randomized controlled trial

**DOI:** 10.1186/s13063-020-4145-2

**Published:** 2020-02-17

**Authors:** Nicolas Bertholet, Elodie Schmutz, Véronique S. Grazioli, Mohamed Faouzi, Jennifer McNeely, Gerhard Gmel, Jean-Bernard Daeppen, John A. Cunningham

**Affiliations:** 1 Addiction Medicine, Lausanne University Hospital and University of Lausanne, Lausanne, Switzerland; 2 Center for Primary Care and Public Health (Unisanté), University of Lausanne, Lausanne, Switzerland; 3 Department of Population Health, Section on Tobacco, Alcohol and Drug Use, New York University (NYU) Grossman School of Medicine, New York, NY 10016 USA; 4 Center for Addiction and Mental Health, Toronto, ON Canada; 5 Department of Psychiatry, University of Toronto, Toronto, ON Canada

## Abstract

**Background:**

Unhealthy alcohol use is a leading cause of morbidity and mortality among young people, including university students. Delivering secondary prevention interventions against unhealthy alcohol use is challenging. Information technology has the potential to reach large parts of the general population. The present study is proposed to test a proactive secondary prevention smartphone-based intervention against unhealthy alcohol use.

**Methods:**

This is a parallel-group, randomized controlled trial (1:1 allocation ratio) among 1696 university students with unhealthy alcohol use, identified by screening and followed up at 3, 6, and 12 months. Participants will be randomized to receive access to a smartphone-based intervention or to a no intervention control condition.

The primary outcome will be self-reported volume of alcohol drunk over the past 30 days, reported as the mean number of standard drinks per week over the past 30 days, measured at 6 months. Secondary outcomes will be number of heavy drinking days over the past 30 days, at 6 months. Additional outcomes will be maximum number of drinks on any day over the past 30 days, alcohol-related consequences (measured using the Short Inventory of Problems (SIP-2R), and academic performance.

**Discussion:**

The aim of this trial is to close the evidence gap on the efficacy of smartphone-based secondary prevention interventions. If proven effective, smartphone-based interventions have the potential to reach a large portion of the population, completing what is available on the Internet.

**Trial registration:**

ISRCTN, 10007691. Registered on 2 December 2019. Recruitment will start in April 2020.

## Introduction

Unhealthy alcohol use is a leading cause of morbidity and mortality among young people, including among university students in whom unhealthy alcohol use is associated with academic impairment, damage to self and others, and institutional costs [45]. In Switzerland and worldwide, a significant proportion of the mortality among young people is attributed to alcohol [13, 35, 36]. Students frequently experience the consequences of unhealthy alcohol use and the consequences tend to increase over time, despite prevention efforts [27]. Students are therefore an appropriate target for secondary prevention interventions aiming at reducing unhealthy alcohol use [1].

**Table 1:** Schedule of enrollment, intervention and assessment (SPIRIT flow diagram)

Smartphone-based secondary prevention intervention for students with unhealthy alcohol use: study protocol of a parallel group randomized controlled trial					
Study periods	Inclusion procedure	Baseline assessment	Follow up		
Time point (month)	0	0	3	6	12
Expected duration (minutes)	3	10	10	10	10
Inclusion criteria	x				
Patient Information and informed consent	x				
Demographics (questionnaire)	x	x			
Primary outcome variables (questionnaires)		x	x	x	x
Secondary outcome variables (questionnaires)		x	x	x	x
Other outcomes of interest (questionnaires)		x	x	x	x
Randomization		x			
Intervention group		Access granted to application			
Application usage data		For intervention group participants, throughout follow-up period			

A challenge for addressing unhealthy alcohol use is that few with unhealthy alcohol use actively seek treatment [18]. Information technology has the potential to reach large parts of the general population [19, 22]. Compared to face-to-face interventions, interventions using information technology have a greater reach, can be more easily implemented, and are more consistent [42]. Numerous electronic interventions using CD-ROM, computers, or the Internet have been developed and tested [31, 50]. Electronic screening and brief intervention are considered effective methods for reducing unhealthy alcohol use [49, 56].

Given its widespread use, the smartphone may be an excellent tool to disseminate interventions, especially among young individuals. In a context in which there is a demand for electronic interventions [30, 57], the development of smartphones offers an opportunity for more proactive interventions, with the potential for multiple contacts, at the user’s convenience, which may help increase the intensity of interventions. Smartphone applications (apps) may help target drinking more specifically by offering interventions at any given time, including in situations in which people are drinking alcohol, for example at parties. Evidence of the efficacy of computer-based or web-based interventions cannot be extrapolated to smartphone applications: notably, computers and smartphones are used differently, which may change their impact. Cohn and colleagues collected data on available smartphone applications and assessed whether these applications incorporated components of evidence-based treatment [12]. They concluded that it is of primary importance to understand for whom and under what conditions mobile technology might be effective, and stressed the importance in the field of research on alcohol of remaining up-to-date with interactive media, to bring alcohol interventions to people who might otherwise not access care [12]. A 2015 review of the available studies on the use of smartphone applications to reduce alcohol use listed six applications [40]. Of these, two were associated with reductions in drinking [26], two were not associated with reductions [24], and two required further examination of possible effects. Evidence on smartphone-based interventions is growing, but more research with robust experimental designs is needed [55]. Even thoughtfully developed applications can be associated with unanticipated adverse effects [24]. In particular, there is a lack of evidence of efficacy of smartphone applications to reduce unhealthy alcohol use among university students [4]. Given the explosion in the development of applications, there is an urgent need for research on the efficacy of smartphone applications to decrease unhealthy alcohol use.

We developed a secondary prevention smartphone application intended for individuals screened with unhealthy alcohol use, tested it in pilot studies, and showed that the application was generally acceptable to users and that its use was associated with reduced alcohol consumption [7, 9].

The present study is proposed to test an intervention developed based on the latter proactive secondary prevention smartphone application, taking into account the limitations observed during the pilot studies [7, 9]. The smartphone application has been modified iteratively, involving members of the study target population (i.e. university students with unhealthy alcohol use identified by screening) with two rounds of face-to-face semi-structured interviews.

The smartphone-based intervention will be tested in a parallel-group, randomized controlled trial (1:1 allocation ratio) among university students with unhealthy alcohol use identified by screening, and followed up at 3, 6, and 12 months. The present project is planned to use the existing infrastructure (emails) of higher education institutions, allowing for an effectiveness study with potential for broad dissemination if positive effects on reductions in drinking are identified.

## Methods

### Research questions

The proposed trial will test the effectiveness of a proactive secondary prevention smartphone-based intervention (application) in a randomized controlled trial among students with unhealthy alcohol use. We will use a no-intervention control condition.

The main research question is: “Is providing access to a smartphone application to students with unhealthy alcohol use effective in reducing drinking (primary outcome), and negative alcohol-related consequences (secondary outcome) or poor academic performances (secondary outcome)?” In addition, as those randomized to receive access to the app will not necessarily use it, we will assess whether using the smartphone application is effective in decreasing drinking. Thus, the secondary research question is: “Is using a smartphone application targeting unhealthy alcohol use effective in reducing drinking?” (Table 1 and Additional file 1).

### Study hypotheses

#### Main hypothesis

Participants randomized to receive access to a smartphone app targeting unhealthy alcohol use will have greater reductions in drinking 6 months later, compared to participants who did not receive access to the app.

#### Secondary hypothesis

Participants who used a smartphone app targeting unhealthy alcohol use will have greater reductions in drinking compared to the no-intervention control condition (i.e. participants randomized to have access to the app and who will actively use it will have greater reduction in drinking than control participants).

### Participants

Participants (*n* = 1696) will be recruited among registered students at four higher education institutions in the French part of Switzerland. Eligible students will have to be 18 years of age or older, screen positive for unhealthy alcohol use (defined as Alcohol Screening Tool Audit (AUDIT)-C score ≥ 4 in men and ≥ 3 in women) [10, 11, 32], own a smartphone, and be willing to complete follow-up assessments. There will be no exclusion criteria except for participation in the qualitative part of the study (semi-structured interviews).

### Recruitment procedure

All students registered at the participating higher education institutions are provided a university/school email address once they are admitted. The participating institution will send an email presenting the study to all students. The study will be presented as a repeated survey of students’ alcohol use, in which participants could be asked to consult prevention material on their smartphone. Because higher education institutions in Switzerland welcome numerous international students, the study will be available to those speaking French and English. Students interested in participating will be directed to a specifically developed study website. On the study website, students interested in participation will complete a screening questionnaire assessing inclusion criteria. The screening will be anonymous and no personal data will be recorded at this point. The questionnaire will include the AUDIT-C, a validated screening questionnaire (including the online version) on unhealthy alcohol use, with differing cut-off scores by gender [10, 32].

Individuals fulfilling the inclusion criteria will receive a description of the study, indicating the follow-up schedule and assurance of confidentiality. Interested potential participants will be directed to complete an online consent form and asked to provide a physical address, an additional email address (at which to be contacted in case they leave the University/school during the study period) and a mobile phone number. They will then receive an email with a unique identifier linking to the baseline questionnaire. Only participants completing the baseline assessment will be included in the trial. Upon completion of the baseline questionnaire, participants will be randomized to one of two groups to receive: (1) assessment + access to personalized smartphone application (intervention condition) or (2) assessment only (control condition). Participants in the control condition will not receive access to the app.

Participants in the intervention group will receive a message containing an ID number. Participants will be asked to download the application and to unlock it using the ID number provided. This will validate the download and “unlock” an incentive. These procedures ensure that participants have a valid phone number, have downloaded the application, and are participating only once in the study. Control group participants will receive a message with an ID number that they will enter on the web to unlock an incentive*.*

### Intervention

The main theoretical bases for the proposed intervention are norms perception and risk perception. According to social norms theory, our perceptions and beliefs of what is “normal” behavior among others influences our own behavior [3, 45–47]. Many of the known Internet-based screening and brief interventions are based on social norms interventions, and contain normative feedback. Research among college students and young individuals has established that a high level of misperception (i.e. the difference between one’s actual behavior and what one thinks is true of others) is associated with higher levels of alcohol use, and that perceived norms are a strong predictor of alcohol use [8, 34, 37, 47]. Consequently, providing information about the actual norms encourages individuals to adopt behaviors that are closer to the population norms (in this case, lower levels of alcohol use) [41].

The second theoretical basis for the intervention is risk perception. Various theories of health-protective behavior have shown the motivational impact of perceived risk. The health belief model, protection motivation theory, the theory of reasoned action and subjective expected utility theory all assume that anticipation of negative health outcomes, and the desire to avoid negative outcome and reduce its impact (perceived benefits) creates motivation for self-protection [51–53]. Studies have shown the mediating role of perceived risk in drinkers’ reactions to interventions for unhealthy alcohol use [20]. A more adequate perception of risk is therefore expected to lead to decreases in drinking or changes in the drinking pattern.

The smartphone application will comprise 6 modules: (1) personalized feedback on self-reported alcohol consumption, with normative feedback, feedback on the calorific content of the reported consumption, and feedback on health risks; (2) blood alcohol content (BAC) computation module; (3) self-monitoring tool; (4) goal-setting tool; (5) designated driver tool; and (6) fact sheets. The intervention content was based on the available literature [2, 14, 25, 29, 43, 44], previous research with electronic interventions conducted by our group [5–7, 9, 16, 21], and the input of users (semi-structured interviews with users, in two consecutive rounds, invited to test a pilot version of the application with modifications made after each round). As the quality of product design is a major predictor of user engagement with electronic interventions, it has been given primary importance and extensively discussed with the participants [2]. In terms of product design, the following elements have been given specific attention: usability, visual design, user engagement, persuasive design (call to action, ongoing feedback and monitoring, data-driven content, rewards), emphasis on a non-judgmental environment and acceptance, and credibility (references for the data used, university hospital logo, references for fact sheets).

### Control condition

The study will use a no-intervention control condition. Given the state of the evidence on smartphone-based interventions, a no-control condition was deemed appropriate. Participants randomized to the control group will be asked to complete the same assessments as in the intervention group but will not be given access to the smartphone app and will not receive any feedback on their alcohol use.

### Outcome measures

The primary outcome will be self-reported volume of alcohol drunk over the past 30 days, reported as the mean number of standard drinks per week over the past 30 days, measured at 6 months. This will be assessed with a validated quantity/frequency measure [48]. It has been demonstrated that electronic self-report of alcohol use is valid and that social desirability bias is limited [15]. Compared to a drinking diary, which is more burdensome to participants, this type of measure has been shown to limit attrition and to be functionally identical to drinking diary methods [17, 54].

Secondary outcomes will be the number of heavy drinking days (i.e. days with five drinks or more consumed by men or four drinks or more by women) over the past 30 days, at 6 months. Additional outcomes will be the maximum number of drinks on any day over the past 30 days, alcohol-related consequences (measured using the Short Inventory of Problems (SIP-2R) [33], and academic performance. Academic performance will be assessed by the following question: “How do you rate your performance in comparison with your fellow students?” (with response options: much worse, worse, similar, better, much better).

These outcomes will be used for the main analysis (primary hypothesis: providing access to the app will be associated with changes in drinking outcomes) and for the per-protocol analysis (secondary hypothesis: accessing the app will be associated with changes in drinking outcomes).

### Randomization

Randomization will be embedded within the study website. Participants will be automatically randomized once the baseline assessment is completed. The generation of the randomized sequence and programming it into the study website will be conducted by information technology programmers who will not have any contact with participants or any involvement in the study implementation. Because the randomization will be embedded within the study website, concealment of allocation will be total. All assessments will be completed electronically. This will ensure blinding of the assessments. In addition, online assessment of alcohol use does not appear to be sensitive to the Hawthorne effect [39]. Assessment questionnaires will be compatible with computer, smartphone, and tablets. For follow-up assessments, participants will receive an email with a unique identifier linking to the follow-up questionnaire.

### Blinding

Participants will be blind to their experimental condition as they will be randomized directly after completing the baseline assessment, with no indication of group allocation. Research staff involved in the study will not be informed of participants’ group allocations.

### Follow up

Participants will complete a follow-up assessment at 3, 6, and 12 months. Follow-up assessments will be conducted electronically. Participants who do not complete the follow-up assessment 3 days after having received the email providing the link to the questionnaire will be sent a first reminder by email. A second reminder will be sent 3 days later. In the case of non-response after two reminders, participants will be contacted by research assistants over the phone (up to five unanswered calls) and by text message (up to three messages). Research assistants will encourage participants to complete the questionnaire online. Once the questionnaire is complete, participants will receive an incentive. Incentives will be electronic coupons (up to 50 Swiss francs (CHF) if all assessments/tasks are completed). This strategy has been used successfully in a previous study with electronic follow up conducted by our group and allowed a high follow-up rate (91% at 6 months among people with unhealthy alcohol use) [5, 6].

### Data collection

All assessments will be conducted electronically and recorded directly on a secure electronic case report form that is independently managed, to ensure data integrity, security, quality, and traceability. The electronic forms will be tested before the study to ensure the quality of data recording. The final trial dataset will be accessible to investigators at the end of the study. Data will be publicly available after publication of the trial results.

### Statistical analyses

The primary analysis will answer the main research question: “Is providing access to a smartphone app effective in reducing drinking compared to a no-intervention control condition?” The primary analysis will be to compare the intervention and control groups at 6 months on the primary outcome measure: the volume of alcohol drunk reported as the mean number of drinks per week over the past 30 days. All available data will be used in an intent-to-treat paradigm (i.e. individuals will be analyzed according to their initial group allocation). Missing values will be handled through a multiple imputation procedure in order to respect the intention-to-treat paradigm [28]. The null hypothesis is that both conditions have comparable effects on the volume of drinking at the 6-month follow up. The alternative hypothesis is that participants in the intervention group (experimental condition: secondary prevention intervention) will report a smaller volume of drinking at the 6-month follow up compared to participants in the control group.

Count models will be used. Because we are expecting that the data will not be normally distributed, an adequate count model will be selected by comparing different count models. From previous studies conducted with the same outcome, we expect a negative binomial regression model to best fit the distribution of the sample [5, 6]. If this is confirmed, the impact of the intervention will be assessed by a random-effects negative binomial model for mean number of drinks per week. The model will specify subject and recruitment site as random effects and intervention and time as fixed effects (with a time * intervention interaction term). The model will be adjusted for age and gender.

#### Secondary outcomes

The same procedure will be applied to secondary outcomes: number of heavy drinking days, maximum number of drinks on any day, SIP-2R score, and academic performance score.

In order to test the secondary hypothesis that participants who used a smartphone app targeting unhealthy alcohol use will have greater reductions in drinking compared to the no-intervention control condition, the analyses will be replicated in the subsample of participants who actively downloaded the application or completed the matching procedure in the control group. Thus, participants randomized to have access to the app and who will actively use it will be compared to those randomized to the no-intervention control condition who completed the matching procedure (entering a personalized code on a website). This analysis is equivalent to a per-protocol analysis.

Analyses will be performed at the completion of data collection. Subgroup analyses will be performed among participants reporting a high level of drinking and potential alcohol use disorder. In order to examine the potential for differences in the efficacy of the application among these participants, we will conduct secondary analyses in subgroups: in men we will conduct secondary analyses among those with baseline AUDIT score > 10 and AUDIT scores > 13 [23]: in women the cut-off points will be AUDIT scores > 6 (optimal sensitivity/specificity for *Diagnostic and statistical manual of mental disorders* (DSM-5) alcohol use disorder) and AUDIT scores > 11) [23].

### Sample size

The sample size computation is based on studies conducted in similar populations, testing the efficacy of electronic interventions. The sample size was computed for the primary analysis: evaluation of intervention effects at 6 months on the number of drinks per week (past 30 days). Considering results reported by our group on an Internet-based brief intervention study conducted in Switzerland [5, 6], the sample size required is 551 individuals per group to demonstrate a significant difference with power of 90% and α type I error of 5%. Considering the potential need to adjust for clustering (recruitment on four sites with hypothetical low intraclass correlation of rho = 0.001) and an attrition rate of 10%, 848 participants per group are needed (771 + 77). A total of 1696 individuals is therefore the minimum number to be included.

### Monitoring

Monitoring will be performed according to the International Council for Harmonisation of Technical Requirements for Pharmaceuticals for Human Use (ICH) Good Clinical Practice (GCP) by the Clinical Trial Unit, Lausanne University Hospital and University of Lausanne. The monitors will follow a monitoring plan to verify that the clinical trial is conducted and that data are generated, documented, and reported in compliance with the protocol, GCP, and the applicable regulatory requirements.

### Ethics

The study, its methods and design, has been approved by the Commission Cantonale d’Ethique de la Recherche sur l’Etre Humain, Vaud (CER-VD) (no 2018–00560).

## Conclusion

This trial is aimed to close the evidence gap on the efficacy of smartphone-based secondary prevention interventions. As of today, evidence on Internet-based interventions for unhealthy alcohol use is available [49], but it is mostly lacking on smartphone interventions, even though this research area has been the focus of more research efforts in recent years [55].

A notable strength is the content and design of our intervention, which is theoretically based and is informed by pilot studies and previous experiences from our group with respect to electronic interventions. From a study design perspective, information technology allows for adequate randomization, concealment of allocation, and blinding. Electronic interventions are also more consistent.

One limitation, the absence of biological measures of alcohol use, should be acknowledged. It was not included for practical reasons, as collecting biological samples in the study sample was not achievable with the available resources. In addition, common biologic measures have poor sensitivity for low-level unhealthy alcohol use. Another potential limitation is the potential for repeated assessments to lead to a reduction in drinking (i.e. assessment reactivity), by initiating a reflection on one’s own alcohol use [38]. Nevertheless, the use of electronic assessments should limit social desirability bias or the Hawthorne effect [39].

### Potential impact and dissemination of results

If proven effective, smartphone-based interventions have the potential to reach a large portion of the population, completing what is available on the Internet. By relying on existing resources within universities or higher education institutions, this trial has the advantage of testing a procedure that could be widely implemented outside a research trial, without significant additional resources (beyond the maintenance of the application). It also targets a population with a high prevalence of unhealthy alcohol use, who suffer significant consequences of unhealthy alcohol use. If proven effective, the application will be made freely available. In accordance with the Swiss National Science Foundation guidelines, the study results will be published in open access publications. Results will also be communicated to the participating universities and higher education institutions.

## Supplementary information

**Additional file 1.**SPIRIT 2013 Checklist: Recommended items to address in a clinical trial protocol and related documents.

## Data Availability

Data will be made available in accordance with the Swiss National Science Foundation open access and data sharing rules and recommendations.
